# Design and development of a novel sliding friction and wear tester with complex working conditions

**DOI:** 10.1038/s41598-021-86451-4

**Published:** 2021-03-25

**Authors:** Jinrong Chai, Zihao Zhou, Cheng Ye, Chen Yao, Guohua Li

**Affiliations:** School of Mechanical Electronic and Information Engineering, China University of Mining and Technology (Beijing), Beijing, 100083 China

**Keywords:** Mechanical engineering, Metals and alloys, Mechanical properties

## Abstract

Serious wear phenomena occur in mining machinery under complex working conditions, and the wear of machine parts is primarily caused by the synergistic effect of adhesive wear, abrasive wear, corrosive wear, etc. However, the existing friction and wear testing equipment cannot be used to carry out wear tests under complex working conditions. To simultaneously meet the test requirements of adhesive wear, abrasive wear, and corrosive wear, a novel sliding friction and wear tester that can simulate complex working conditions was developed in the present research. The tester is composed of a loading mechanism, a speed-regulating mechanism, a corrosion chamber, and a control and display system. Wear tests of the middle plate of a scraper conveyor, a key equipment of coal mining, were carried out to verify the consistency and effectiveness of the tester. The test results were consistent, and those under the same test conditions were similar with a maximum standard deviation of 2.4 mg. The wear condition of the middle plate specimens was close to the actual wear condition of the middle plate. Moreover, the surfaces of the middle plate specimens after grinding exhibited obvious adhesive, abrasive, and corrosive wear characteristics, and the wear degrees of the specimens under the same test conditions were similar. The quality loss of the middle plate specimens was found to increase with the increase of coal gangue percentage, and the main wear mechanism was the synergistic action of abrasive, adhesive, and corrosive wear.

## Introduction

Mining machinery often works in complex environment characterized by corrosion, abrasive, damp, high temperature, and other harsh conditions; thus, severe wear often occurs in this machinery. The wear of machinery components is caused by the combined action of multiple wear types, such as abrasion, adhesion, corrosion, etc.^1–5^. Serious wear phenomena are particularly obvious on the middle plate of the scraper conveyor, the key equipment of coal mining^6–13^. The general testing equipment for the research of the wear of mining machinery are the MLS-225 wet abrasive wear tester, the MLG-130 dry abrasive wear tester and the MLD-10 impact abrasive wear tester^9,14,15^. In addition to general equipment, researchers often develop special friction and wear tester according to the actual working conditions. For instance, Chokecherry et al.^16^ designed and manufactured a test apparatus that combines the principle of a pin-disk test and three-electrode electrochemical corrosion tests. This tester meets the requirements of evaluating the material wear resistance in marine environments. Li et al.^17^ developed an abrasive wear testing machine for a mining scraper conveyor that can simulate the wear of two friction pairs, such as the scraper and the middle plate, or the scraper and the groove. A novel sliding friction setup was developed, which was performed by a single diamond grain at nanoscale depth of cut and speeds of m/s^18^. The sliding speeds are three to six orders magnitude higher than conventional mm/s and μ m/s for nanoscratching and sliding^19,20^. This method opens a new pathway to investigate the fundamental mechanisms of abrasive wear. Under the guidance of the method, novel diamond wheels and machining methods were developed^21,22^. Zhang et al.^23^ studied the corrosive wear behavior of the P110S casing steel in NaCl solutions with different pH values via the use of a self-made corrosive wear tester. Li et al.^24^ modified the MLD-10 impact abrasive wear tester. Cora et al.^25^ developed a novel wear evaluation system for stamping die materials to better simulate the working conditions of the die and evaluate the wear performance of the materials. Kurčík et al.^26^ designed a tester to assess the wear of a vehicle braking system. However, these existing friction and wear testers do not have the function of performing corrosive-abrasive wear tests under the condition of sliding friction, and cannot meet the testing requirements under complex working conditions. Therefore, it is necessary to develop a novel friction and wear testing machine to meet the requirements of sliding friction and wear tests under complex working conditions.

To this end, according to the complicated sliding friction working conditions and the actual wear situation, a novel sliding friction and wear tester for use under complicated working conditions was developed in this study. The tester can be used to carry out sliding friction and wear tests under the joint or separate action of abrasive, adhesive, and corrosive wear. The design and construction of the novel tester are discussed in detail. Moreover, wear tests of the middle plate of a scraper conveyor, a key equipment of coal mining, were carried out to verify the consistency and effectiveness of the proposed tester.

## Design and construction

### Design

**Figure 1:**
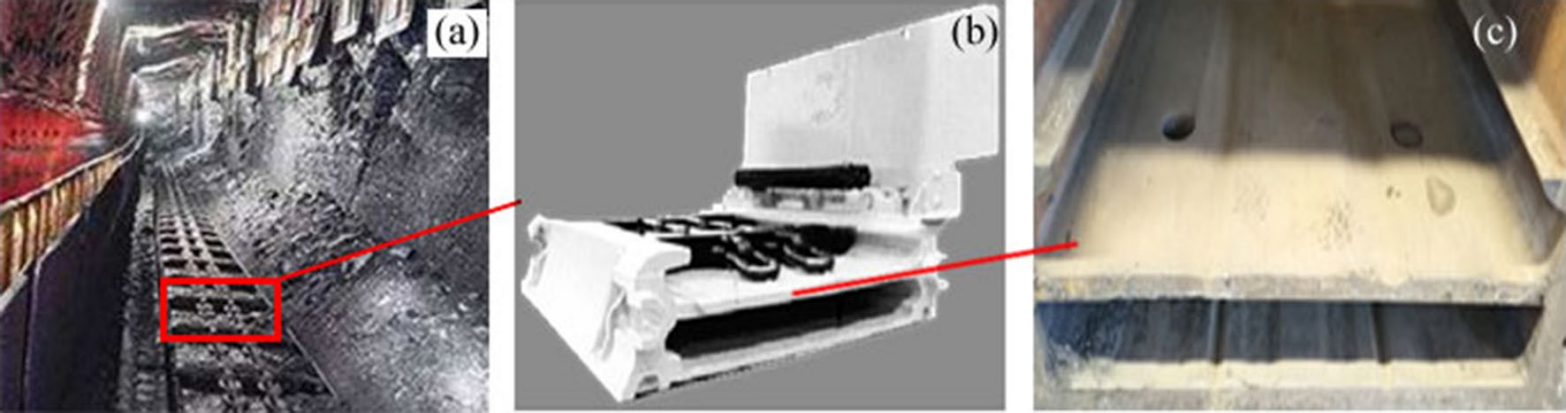
The actual wear of a scraper conveyor: (**a**) the working environment of the scraper conveyor; (**b**) the single middle trough; (**c**) the wear of the middle plate.

**Figure 2:**
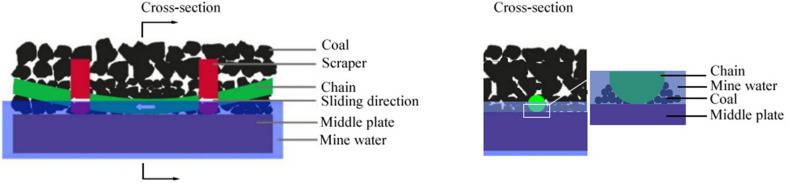
The friction and wear pattern of a scraper conveyor.

**Figure 3:**
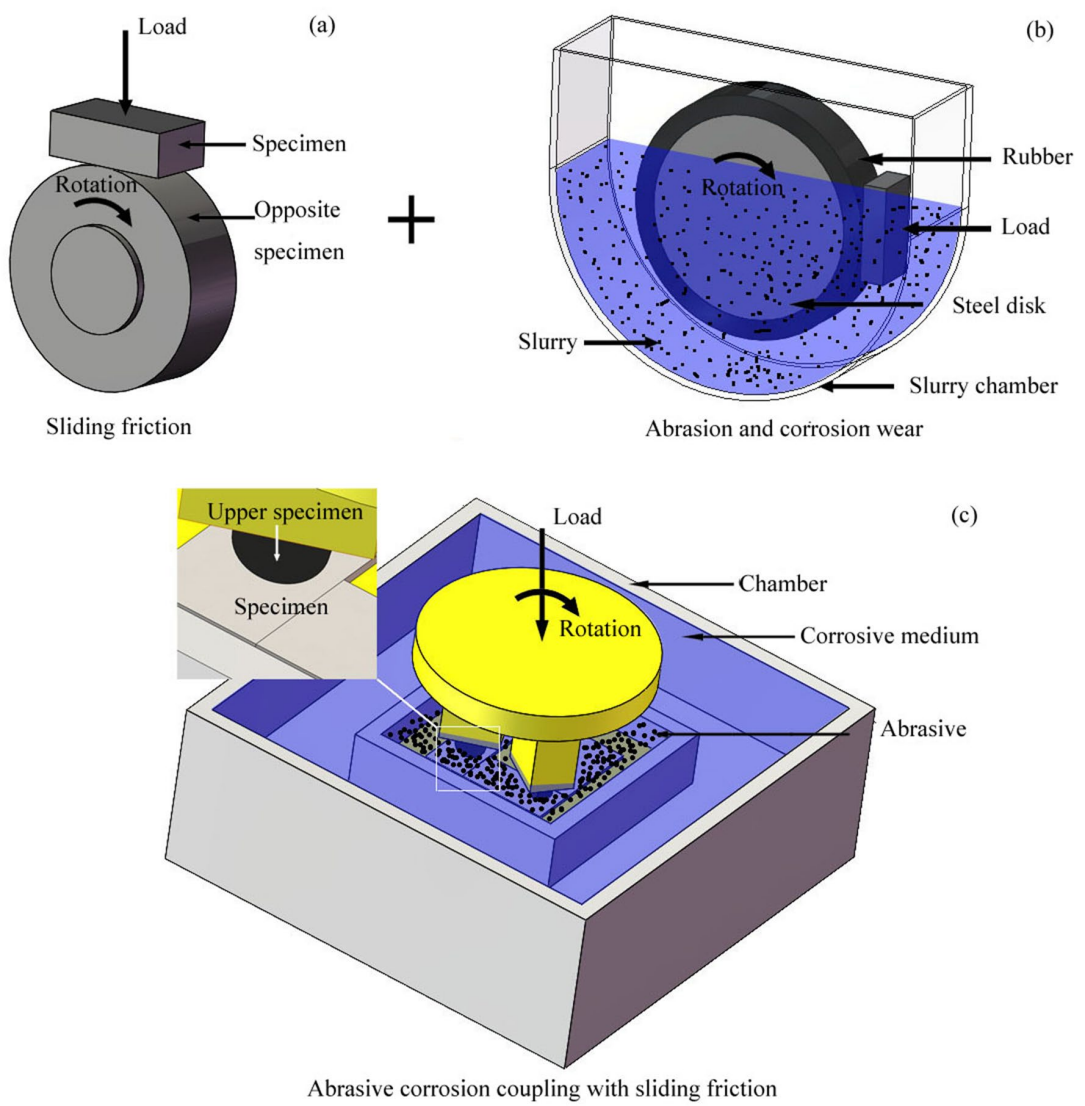
The schematic diagram of the design concept of a wear tester that can simulate complex working conditions: (**a**) the principle of sliding friction and the wear tester; (**b**) the principle of a wet abrasive wear tester; (**c**) the principle of a wear tester that can simulate complex working conditions.

The components of mining machinery working under complex conditions often suffer serious wear. In this study, the wear of the middle plate of a scraper conveyor was taken as an example to analyze the actual working conditions, as shown in Fig. 1. According to the working condition of the scraper conveyor, with the participation of coal particles and mine water, sliding friction occurs between the middle plate and the chain, and between the middle plate and the scraper. The wear of the middle plate is the result of the synergistic effect of abrasive, adhesive, and corrosive wear. The wear diagram of the scraper conveyor is presented in Fig. 2. As shown in the figure, simulated working condition tests of the middle plate material must be carried out under the conditions of sliding friction with the participation of abrasive coal particles and mine water. Therefore, the design of the tester considers the combination of all three conditions, as shown in Fig. 3.

### Structure and control/display system

**Figure 4:**
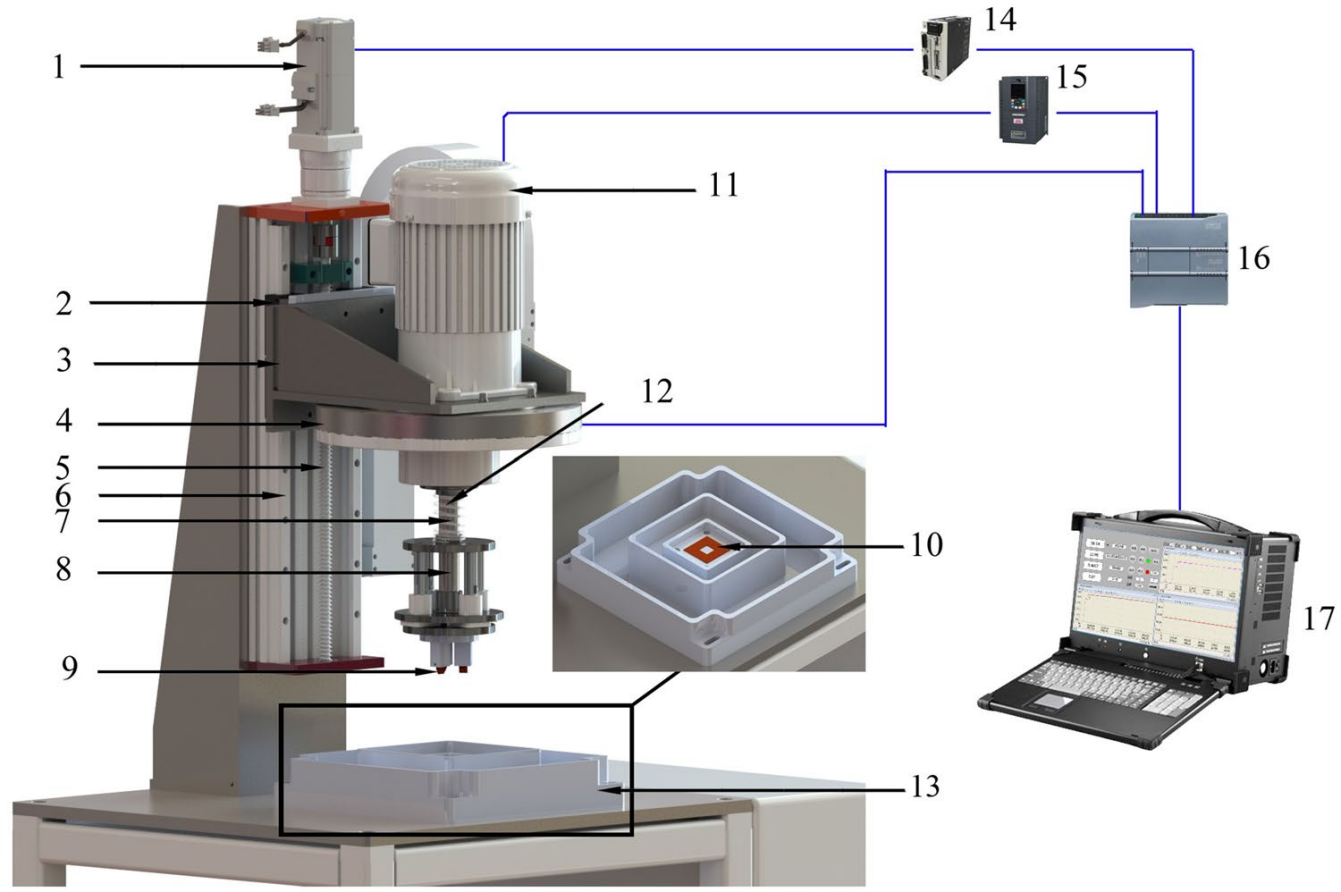
The schematic diagram of the sliding friction and wear tester that can simulate complex working conditions. 1. Servo motor; 2. Slider; 3. Loading flange; 4. Positive-pressure detection mechanism; 5. Screw; 6. Linear guide rail; 7. Spring; 8. Grinding head mechanism; 9. Upper specimens; 10. Specimens; 11. Frequency conversion motor; 12. Spindle; 13. Corrosion chamber; 14. Servo driver; 15. Frequency converter; 16. Programmable logic controller; 17. Principal computer.

Figure 4 presents the structural diagram of the tester. The tester consists of four parts, namely a loading mechanism, a speed-regulating mechanism, a corrosion chamber, and a control and display system. The loading mechanism consists of a servo motor, a slider, a loading flange, a positive-pressure detection mechanism, a screw, a linear guide rail, a spring, a grinding head mechanism, upper specimens, and specimens. The speed-regulating mechanism is composed of a frequency conversion motor and a spindle. The control and display system consists of electrical control system and display system, and the main components of which include a servo driver, frequency converter, programmable logic controller (PLC), and principal computer (PC). The corrosion system must remain as stationary as possible with little or no movement and minimum vibration^15^. Therefore, the corrosion chamber was designed to be static, and the specimen is fixed in the corrosion chamber. The upper specimen rotates and moves vertically. Relative sliding is realized by the rotation of the upper specimen, and loading is realized by moving the upper specimen.

During loading, the servo motor rotates the screw, which drives the slider installed on the linear guide rail to move downward. The loading flange is attached to the slider and moves downward with it. The grinding head mechanism is attached to the loading flange and moves downward with it. The spring is installed between the loading flange and the grinding head mechanism. The upper specimen is installed on the grinding head mechanism. When the upper specimen contacts the specimen, it continues to move downward to compress the spring and produce a spring force, which is applied to the surface of the specimen.

The relative speed of the friction pair is adjusted by adjusting the speed of the frequency conversion motor. The output shaft of the motor is connected to the spindle by coupling, and the spindle is connected to the grinding head mechanism. The torque output of the frequency conversion motor is transferred to the grinding head mechanism via coupling and the spindle to cause the relative sliding of the upper specimen and specimen.

The positive pressure between the surfaces of the friction pairs is transmitted to the PLC via the positive-pressure detection mechanism. The PLC controls the rotation of the servo motor according to the set test load value to control the displacement of the loading mechanism and adjust the positive pressure. The relative sliding speed between the surfaces of the friction pairs is transmitted to the PLC via the frequency converter. The PLC controls the speed of the variable-frequency motor according to the set test speed value to adjust the relative sliding speed of the friction pairs. The positive pressure, relative sliding speed, and torque of the variable-frequency motor are transmitted from the PLC to the PC and displayed in real time. The friction value is calculated from the motor torque using a specific formula. The coefficient of friction is the ratio of the friction to the positive pressure. The friction and friction coefficient are displayed in real time by the PC.

### Structure design of the friction pair and the selection of the corrosion medium

**Figure 5:**
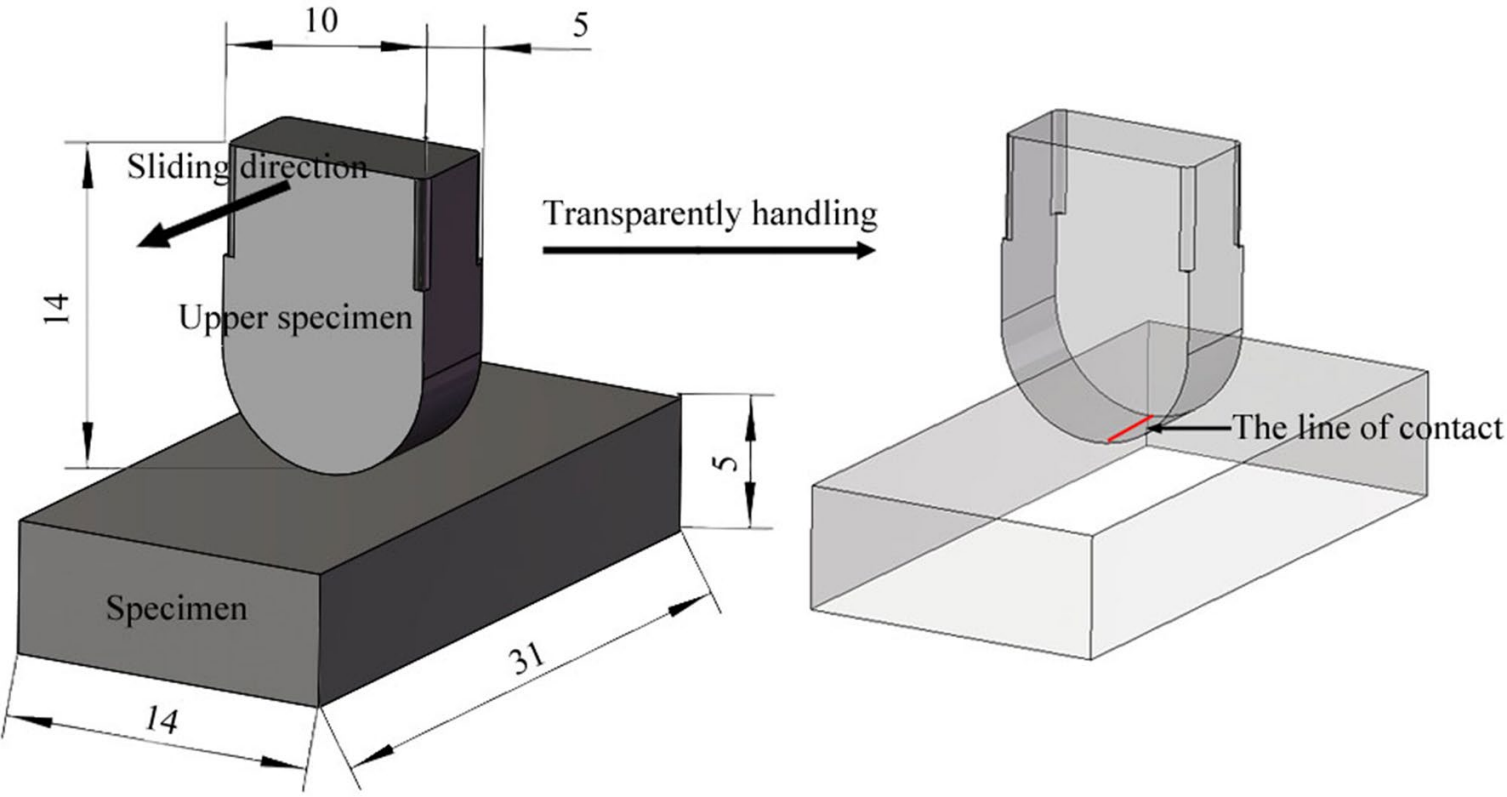
The schematic diagram of the sample structure.

**Figure 6:**
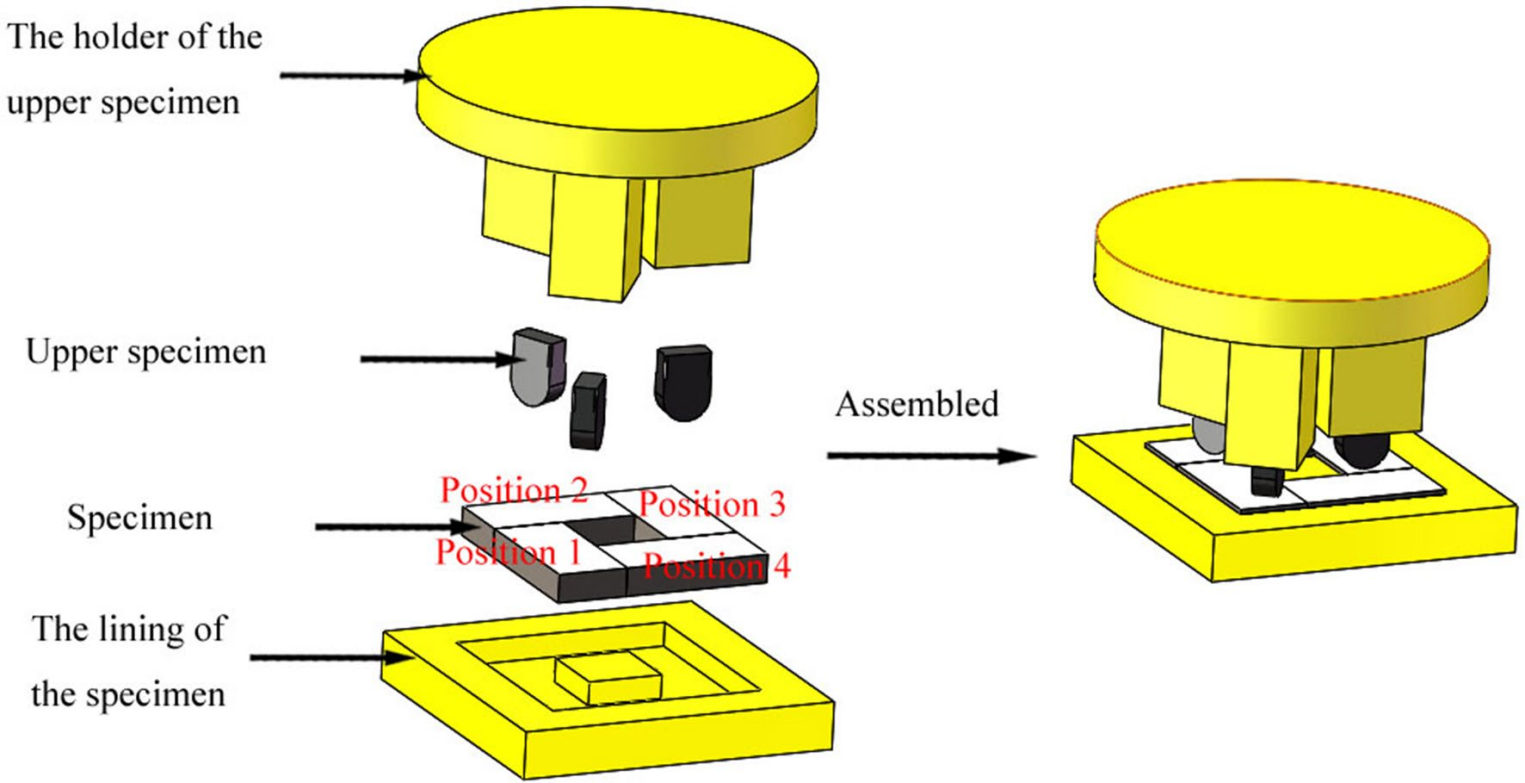
The sample installation diagram.

The novel friction and wear tester can be used to carry out tests of different contact forms, such as point, line, and surface contact, by changing the structure of the upper specimen. The contact form of the friction pair should be selected according to the actual working conditions. In this work, the structure of the friction pair was designed by taking the middle plate and chain of a scraper conveyor as an example. By observing the cross-sectional diagram of the friction pairs of the scraper conveyor presented in Fig. 2, it can be seen that the contact form between the middle plate and the chain is line contact. Therefore, the structure of the samples was designed as follows: the upper specimen was a block structure that was 14 mm long, 10 mm wide, and 5 mm thick with a curved bottom; the specimen was also a block structure that was 31 mm long, 14 mm wide, and 5 mm thick, as shown in Fig. 5. There are four test positions on the tester. To improve the convenience of the test installation, the lining of the specimen and the holder of the upper specimen were designed, and their material was chosen as corrosion-resistant polypropylene. The sample installation diagram is illustrated in Fig. 6. The material of the friction pair should be selected according to the actual working conditions.

The corrosion medium should be prepared according to the actual working conditions, such as the water quality, chemical composition of the water, etc.

## Wear test method

### Materials

**Figure 7:**
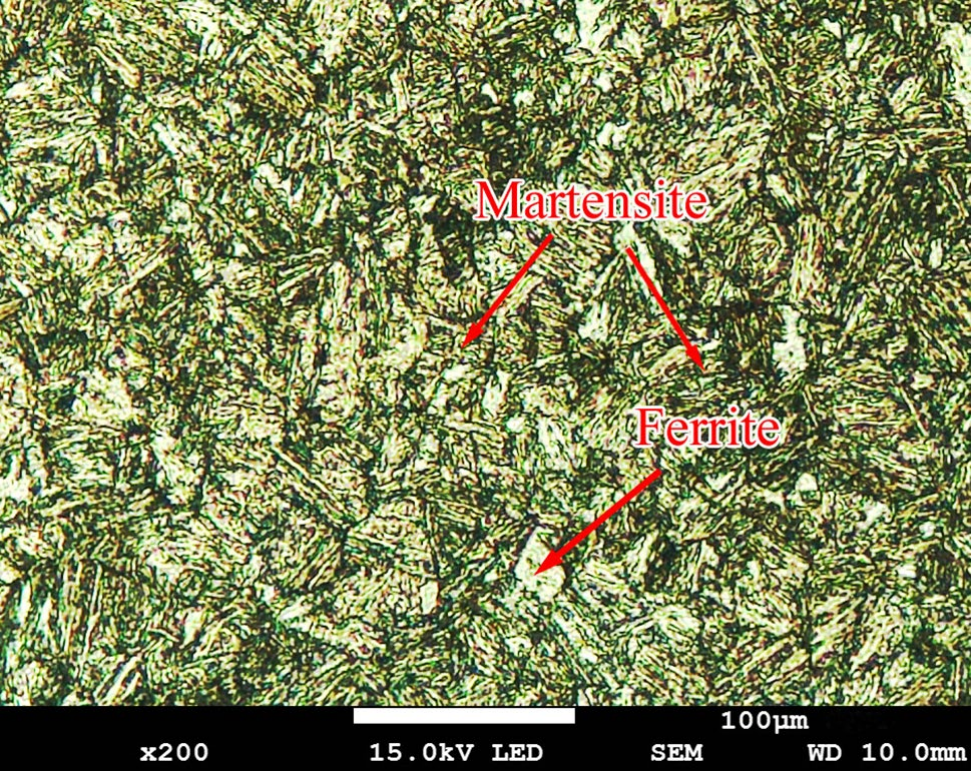
The optical micrograph image of NM400, revealing martensite (dark region) and ferrite (white region).

**Figure 8:**
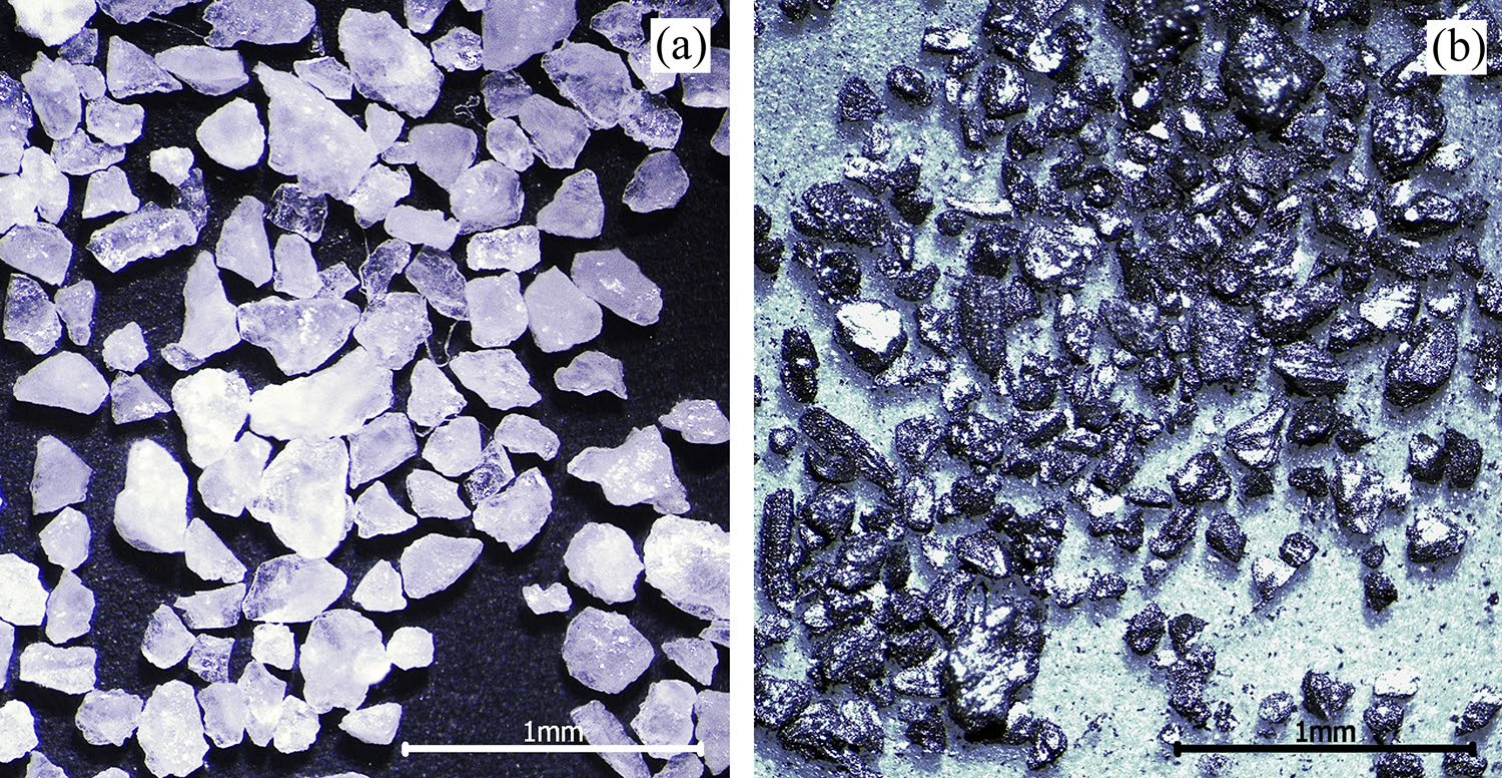
The morphologies of the abrasives used in the wear test: (**a**) quartz sand; (**b**) coal.

The tester can simulate a variety of complex working conditions. Wear tests were carried out to simulate the actual working conditions of the middle plate of a scraper conveyor. The samples were processed with a common scraper conveyor material, namely NM400 (Wuhan Iron and Steel Company), and the upper specimen was 40Cr steel. The material composition is reported in Table 1. The microstructure of NM400 consists of martensite in the dark region and ferrite in the bright region, as shown in Fig. 7. The average hardness of NM400 is 37.2 HRC. The corrosion solution was simulated mine water composed of CaSO_4_, MgSO_4_, Na_2_SO_4_, KCl, NaCl, CaCl, H_2_SO_4_ solution (c(H^+^) = 0.2 mol/L), and distilled water, and the concentration of each substance is reported in Table 2. To simulate the working conditions, the abrasive was created by mixing coal and quartz sand. The coal was anthracite with a particle size of 60–80 mesh, and the particle size of the quartz sand was 60–80 mesh. The abrasive morphology is presented in Fig. 8.

**Table 1:** The chemical composition of NM400 (wt.%).

Chemical elements	C	Si	Mn	P	S	Ti	Cr	Mo	B
Mass fraction(%)	0.160	0.230	1.420	0.008	0.003	0.012	0.170	0.020	0.001

**Table 2:** The concentrations of substances in the simulated mine water.

Substance	CaSO_4_ (mg/L)	MgSO_4_ (mg/L)	Na_2_SO_4_ (mg/L)	KCl (mg/L)	NaCl (mg/L)	CaCl (mg/L)	H_2_SO_4_ (c(H^+^) = 0.2 mol/L) (mL)
Concentration	70	50	40	12	141	10	200

### Test method

#### Consistency and effectiveness verification of the test results

To verify the consistency and effectiveness of the tester, verification tests were carried out under two experimental conditions, as reported in Table 3. Taking a 30% coal gangue as an example, the percentage of coal gangue can be defined as follows: for every 100 g of the abrasive, coal accounted for 70 g and quartz sand accounted for 30 g. Moreover, the test time was set as 60 min. Before and after the test, the specimens were washed with alcohol and weighed on an electronic scale with an accuracy of 0.1 mg to calculate weight loss of the sample due to wear. The average weight loss of four test positions was taken as the result of each test. Each experiment was repeated five times. The wear consistency of the specimen at every position was analyzed. The consistency of results of the five tests was analyzed. After the wear test, a JSM-7800 scanning electron microscope and a LeicaSCAN DCM8 confocal microscopy were utilized to analyze the microstructures of the wear surfaces of the specimens to verify the validity of the results.

**Table 3:** The experimental conditions of the consistency verification tests.

Test serial number	Load (N)	Rotation speed (r/min)	Percentage of coal gangue (%)	pH value of the simulated mine water
1	150.0	100.0	30.0	3.5
2	150.0	100.0	50.0	3.5

#### Wear tests under different working conditions

The percentage of coal gangue is is an important index by which to evaluate the working condition of coal mining machinery, and it has an obvious influence on the wear of the middle plate. Therefore, wear tests were carried out with different percentage of coal gangue were carried out. Three experiments were performed, each of which was repeated three times. The experimental conditions are reported in Table 4, and the other conditions were the same as those described in “Consistency and effectiveness verification of the test results”.

**Table 4:** The experimental conditions of wear tests with different percentage of coal gangue.

Test serial number	Load (N)	Rotation speed (r/min)	Percentage of coal gangue (%)	pH value of the simulated mine water
1	150.0	100.0	30.0	3.5
2	150.0	100.0	50.0	3.5
3	150.0	100.0	70.0	3.5

## Results and discussion

### Consistency analysis of the weight loss at the test positions

Table 5 presents the weight loss at each test position. The consistency of wear at each test position was evaluated by the standard deviation; the smaller the standard deviation, the higher the consistency of wear at each test position. The test positions are indicated in Fig. 6. The minimum, maximum, and average standard deviations were respectively 2.1, 5.8, and 3.38 mg; thus, the wear conditions of each test position exhibited high consistency.

**Table 5:** The wear of specimens at different positions.

Test position	Weight loss in the five repetitions of Test #1 (mg)					Weight loss in the five repetitions of Test #2 (mg)				
	1	2	3	4	5	1	2	3	4	5
1	29.3	27.2	21.6	27.9	23.7	40.7	40.8	42.8	42.1	39.2
2	26.8	25.1	21.2	23.3	25.6	38.6	43.6	44.7	39.9	39.4
3	23.4	29.5	26.6	26.7	26.7	41.0	40.5	43.4	40.9	43.3
4	28.4	21.7	25.2	25.1	27.4	38.9	36.3	40.2	43.1	43.2
Average value	27.0	25.9	23.7	25.8	25.9	39.8	40.3	42.8	41.5	41.3
Standard deviation	4.5	5.8	4.6	3.5	2.8	2.1	5.2	3.3	2.5	4.0

### Consistency analysis of the weight loss during the repeated tests

Table 6 reports the weight loss during the repeated tests, from which it can be seen that the maximum standard deviation was 2.4 mg. Moreover, the concentration of the test data was high, which indicates that the results of the testing machine exhibited good consistency.

**Table 6:** The wear of specimens during repeated tests.

The serial number of repeated tests	Weight loss (mg)	
	Test #1	Test #2
1	27.0	39.8
2	25.9	40.3
3	23.7	42.8
4	25.8	41.5
5	25.9	41.3
Average value	25.6	41.1
Standard deviation	2.4	2.3

### Validation of the test results

**Figure 9:**
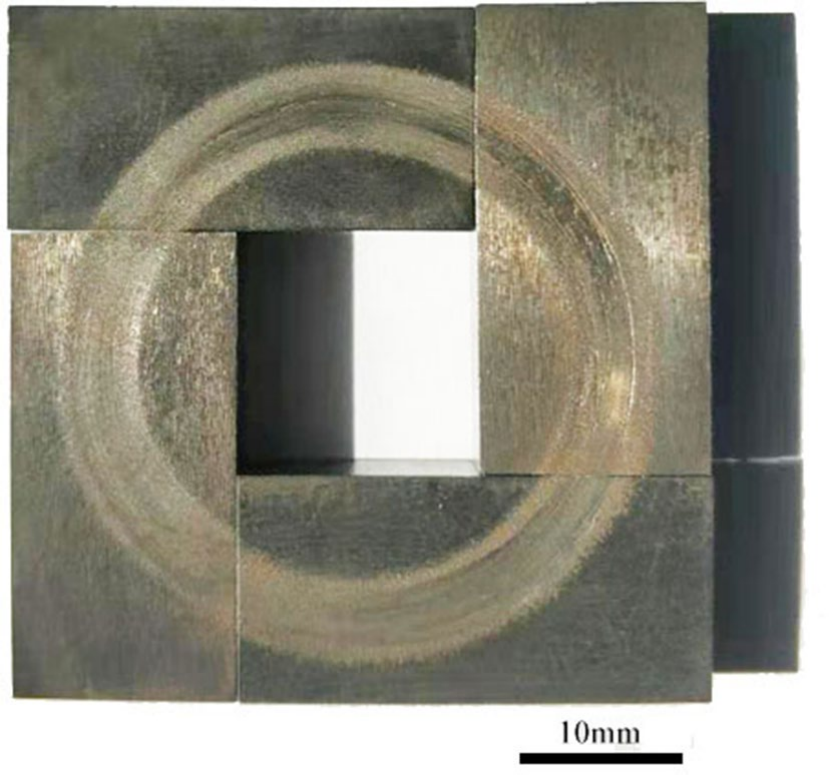
The worn surfaces of specimens under the experimental conditions of 30% coal gangue, a load of 150 N, a rotation speed of 100 r/min, and simulated mine water with a pH of 3.5.

Figure 9 presents the wear trace of the specimen under the test conditions of a load of 150 N, a rotation speed of 100 r/min, 30% coal gangue, and simulated mine water with a pH of 3.5, which are similar to the actual wear conditions of the middle plate presented in Fig. 1c.

**Figure 10:**
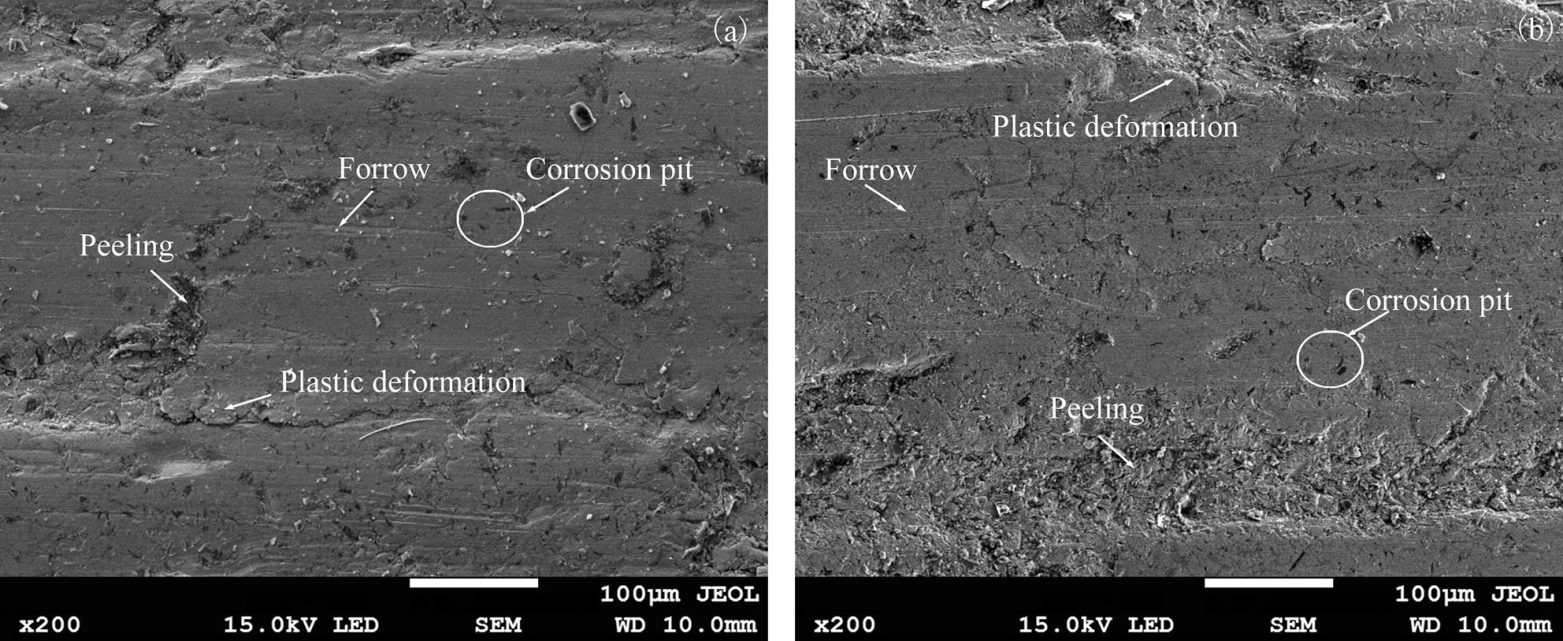
Micrographs of the worn surfaces of NM400 tested under the conditions of 30% coal gangue, a load of 150 N, a rotation speed of 100 r/min, and simulated mine water with a pH of 3.5: (**a**) the first experiment; (**b**) the second experiment.

Figure 10 shows the wear micrograph of the first and second repeated tests of the specimen under the conditions of load 150 N, speed 100 r/min, 30% coal gangue, and mine water with a pH of 3.5. The sliding direction was horizontal. By comparing Fig. 10a and 10b, it can be discerned that the wear degrees of specimens under the same test conditions were largely the same. The wear surface of the specimen exhibited furrows, material peeling pits, and corrosion pits. The furrows were generated by the ploughing action of the hard abrasive. The peeling pits and corrosion pits were formed when the surface material reacted to form softer compounds and then fell off from the material during wear and corrosion. The wear mechanism was the joint action of abrasive, adhesive, and corrosive wear. The tester can therefore be used to carry out tests of abrasive–adhesive–corrosive wear, and to evaluate the wear resistance of the medium plate under complex working conditions.

### Analysis of the influence of the percentage of coal gangue on abrasive–adhesive–corrosion wear

**Figure 11:**
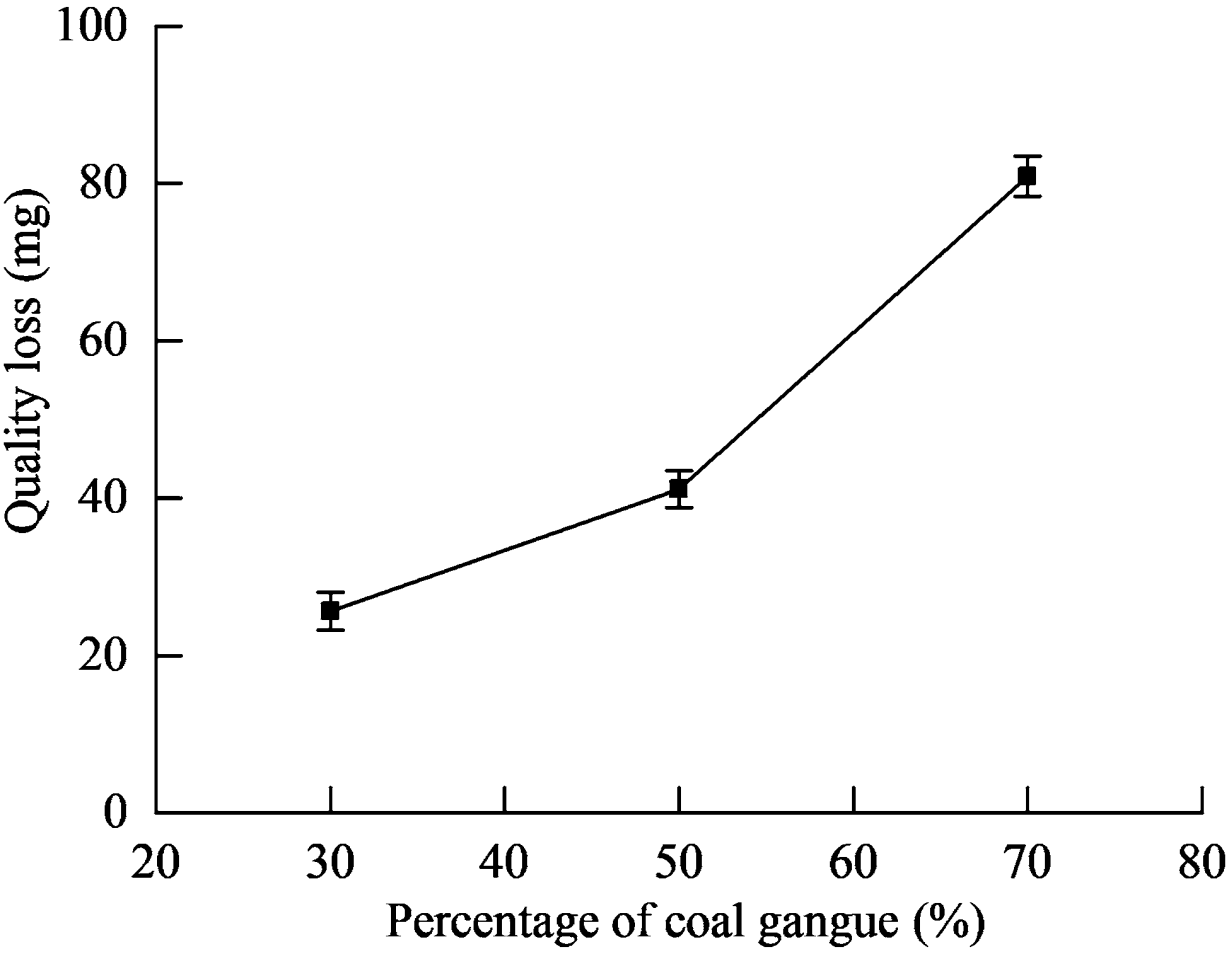
The relationship between the percentage of coal gangue and quality loss.

**Figure 12:**
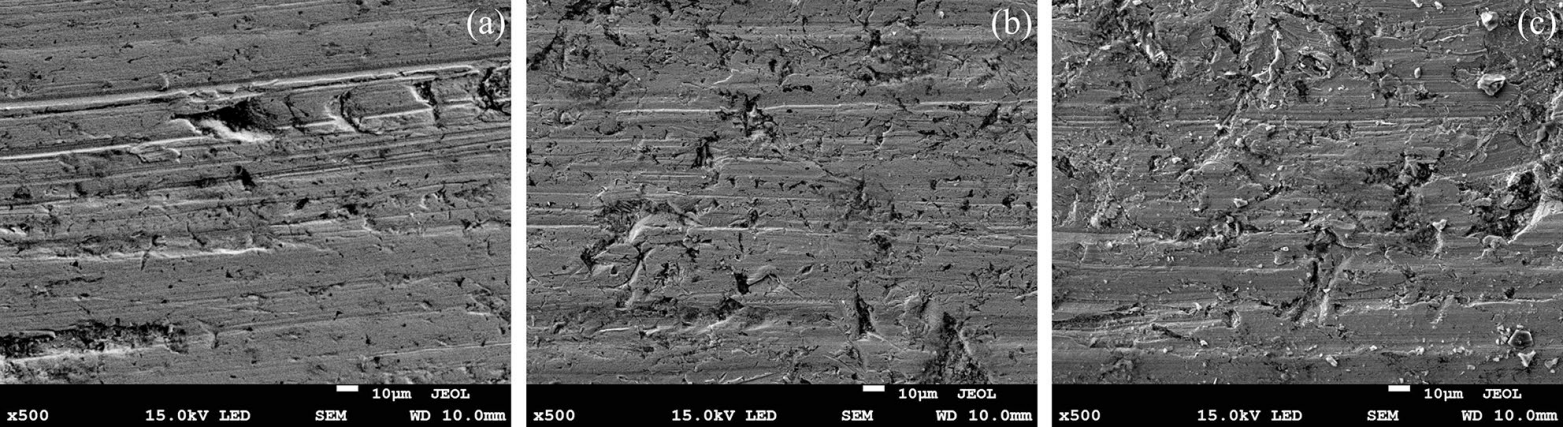
The wear micromorphologies of specimens with different percentage of coal gangue: (**a**) 30%; (**b**) 50%; (**c**) 70%.

**Figure 13:**
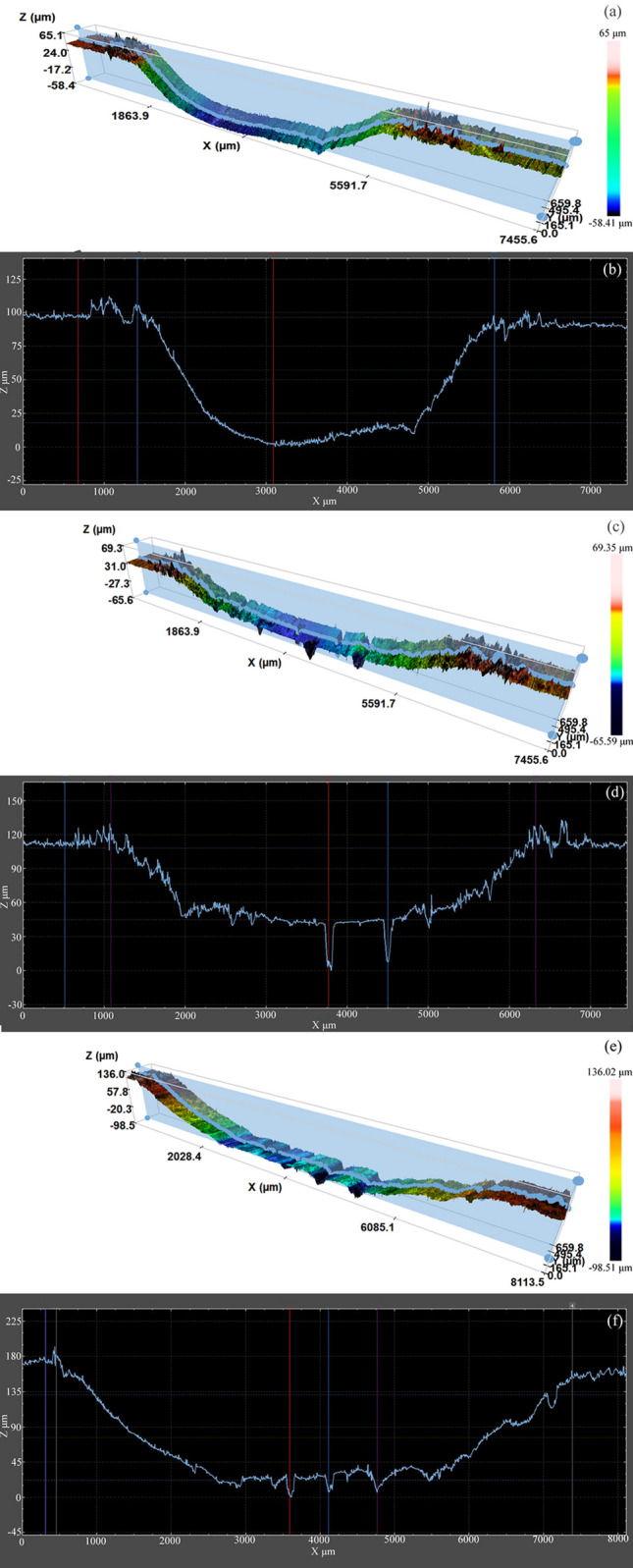
The surface topographies of specimens with different percentage of coal gangue: (**a**) and (**b**) is 30%; (**c**,**d**) is 50%; (**e**,**f**) is 70%.

Figure 11 displays the relationship between the coal gangue content and quality loss. The quality loss of all samples was found to increase with the increase in the percentage of coal gangue. Figures 12a–c respectively present the wear micrographs of the specimens with 30%, 50%, and 70% coal gangue. When the percentage of coal gangue is 30%, the wear surface of NM400 exhibited furrows caused by the ploughing action of hard particles, material peeling pits caused by the combined action of mechanical action and chemical reaction, and corrosion pits caused by chemical reaction. When the percentage of coal gangue is 50%, the number of furrows on the NM400 wear surface increased, the peeling pits and corrosion pits became larger, and the plastic deformation was obvious. When the percentage of coal gangue is 70%, the number of furrows continued to increase, the peeling pits and corrosion pits were connected to form a large area of shedding, and the plastic deformation was intensified. Thus, the increase of the percentage of coal gangue intensified the abrasive wear of NM400. Moreover, under the sliding friction and wear conditions in the corrosive environment, the increase of the effect of hard particles on the material surface caused the pressure and temperature on the material surface to increase, thereby aggravating the adhesive and corrosive wear of the material. Therefore, the wear mechanism of NM400 was found to be the synergistic action of abrasive, adhesive, and corrosive wear. Figures 13a–c respectively show the surface topographies of specimens with 30%, 50%, and 70% coal gangue percentage. With the increase of the coal gangue percentage, the wear surface was found to become increasingly rougher, and the width and depth of the wear marks increased. These phenomena are consistent with the actual situation of the middle plate.

## Conclusion

In this study, a novel sliding friction and wear tester that can simulate complex working conditions was developed, and its consistency and effectiveness were verified. The conclusions of this research can be drawn as follows.

1. The wear condition of each test position of the tester is consistent. The minimum, maximum, and average standard deviations of the specimen’s quality loss at each test position were found to be 2.1, 5.8, and 3.38 mg, respectively.
2. The wear conditions of the tester after repeated tests are consistent. The maximum standard deviation of the specimen’s quality loss during each test was found to be 2.4 mg.
3. The test results of the tester under complex working conditions are effective. The grinding morphology of the specimen was found to be similar to the actual wear morphology of the middle plate of a scraper conveyor, and the wear degrees of the specimen under the same test conditions were largely the same. The wear of NM400 was found to increase with the increase of the percentage of coal gangue. The wear mechanism of NM400 was found to be the synergistic effect of abrasive, adhesive, and corrosive wear. Thus, the proposed testing machine can effectively simulate complex working conditions.
